# Multicopper Oxidases in *Saccharomyces cerevisiae* and Human Pathogenic Fungi

**DOI:** 10.3390/jof6020056

**Published:** 2020-04-27

**Authors:** Tanmoy Chakraborty, Renáta Tóth, Joshua D. Nosanchuk, Attila Gácser

**Affiliations:** 1 Department of Microbiology, University of Szeged, Kozep fasor, 6726 Szeged, Hungary; tanmoy.microbiology@gmail.com (T.C.); renata.toth@bio.u-szeged.hu (R.T.); 2 Departments of Medicine and Microbiology and Immunology, Albert Einstein College of Medicine, New York, NY 10461, USA; josh.nosanchuk@einsteinmed.org; 3 MTA-SZTE Lendület Mycobiome Research Group, University of Szeged, 6726 Szeged, Hungary

**Keywords:** multicopper oxidases, laccase, virulence, iron uptake, pathogenic fungi

## Abstract

Multicopper oxidases (MCOs) are produced by microscopic and macroscopic fungal species and are involved in various physiological processes such as morphogenesis, lignin degradation, and defense mechanisms to stress inducing environmental conditions as well as fungal virulence. This review will summarize our current understanding regarding the functions of MCOs present in *Saccharomyces cerevisiae* and in different human fungal pathogens. Of the two main MCO groups, the first group of MCOs is involved in iron homoeostasis and the second includes laccases. This review will also discuss their role in the pathogenesis of human fungal pathogens.

## 1. Introduction

Multicopper oxidases (MCOs) are blue copper containing proteins and they generally have multiple copper atoms (1 to 6) per molecule [1,2]. Diverse enzymes belong to this family of proteins that include laccases, ferroxidases, ascorbate oxidase, bilirubin oxidase and laccase-like MCOs [3]. MCOs are produced by both prokaryotic as well as various eukaryotic organisms. MCOs consist of four enzyme families: laccases (EC 1.10.3.2), ascorbate oxidases (EC 1.10.3.3), ferroxidases (EC 1.16.3.1), and ceruloplasmin (EC 1.16.3.1) [4]. Detailed analyses of 350 different MCOs were used to classify them in 10 super families, which are: (A) basidiomycete laccases, (B) ascomycete laccases, (C) insect laccases, (D) fungal pigment MCOs, (E) fungal ferroxidases, (F) fungal and plant ascorbate oxidases, (G) plant laccase-like MCOs, (H) copper resistance proteins (CopA), (I) bilirubin oxidases, and (J) copper efflux (CueO) proteins [2]. All MCOs can oxidize aromatic compounds, and can be divided into two functional classes [5]. The first group consists of laccases and laccase-like enzymes (e.g., laccase-like polyphenol oxidases in *Arabidopsis thaliana* encoded by *TT10* gene) [6], favoring oxidation of organic substrates over metal ions. The second group oxidizes metal ions (Fe (II), Cu (I) and/or Mn (II)) with high efficiency, and these MCOs are generally referred to as metal oxidases [7]. These enzymes catalyze oxidation of substrates with a concomitant reduction of molecular oxygen to water. Detailed spectroscopic and X-ray crystallographic analyses of electronic and geometric structure of the active site of MCOs reveal that the catalytic center responsible for these reactions is comprised of four or more copper atoms, leading to three T types which are T1 (blue copper), T2 (normal copper), and T3 (binuclear copper center). Due to the role of Cu in a diverse array of biological oxidation–reduction reactions as a cofactor, MCOs also have a high redox potential in a wide variety of reactions [8,9]. The substrate undergoes oxidation in the T1 center having the His-Cys-His (characteristic of MCOs) sequence, where the Cu atom is bound by two histidine residues and one cysteine forming a distorted trigonal pyramid structure. The unique structure of the MCO enzymes allows the binding and reduction of molecular oxygen into water [10]. Most MCOs contain around 500 amino acid residues and display a β-sheet structure, forming a Greek Key motif [11]. The three main steps of MCO catalytic mechanisms include: (1) T1 Cu site reduction through the capture of an electron from the oxidized substrate, (2) electron transfer from the T1 site to the trinuclear Cu cluster, and (3) O_2_ reduction to two H_2_O molecules. Structurally, laccases belong to the group of multicopper blue proteins that contain two domains, whereas yeast ferroxidases are six domain-containing multicopper blue proteins, similarly to human ceruloplasmin. An X-ray crystallography study showed that laccases do not undergo drastic conformational changes after losing their Cu atom, contrary to ceruloplasmin [12].

The influence of several laccases and ferroxidases on pathogenesis has been reported in various plant pathogenic fungi such as *Magnaporthe grisea*, *Ophiostoma novo-ulmi*, *Gaeumannomyces graminis,* and *Ustilago maydis* [13,14,15,16]. Ferroxidases present in the plant pathogen *Colletotrichum graminicola* complement the growth defect of ferroxidase mutants in *Saccharomyces cerevisae* [17]. In the white rot fungus, *Phanerochaete chrysosporium,* a new class of MCOs has been discovered which is structurally different from conventional fungal laccases [18]. A recent study also revealed gene duplication events, the evolutionary history of MCO genes and the importance of horizontal gene transfer in coprophilous and non-coprophilous fungi [19]. The presence of various MCOs and their importance is also reported in diverse human fungal pathogens. Specifically, ferroxidases involved in iron oxidation and laccases involved in pigmentation and their association with virulence have been reported by different research groups. The focus of the current review is to provide an updated knowledge of ferroxidases in *S. cerevisiae* and ferroxidases as well as laccase in different human pathogenic fungi and their role in iron metabolism and pathogenesis development.

## 2. Multicopper Oxidases in *Saccharomyces cerevisiae*

**Figure 1:**
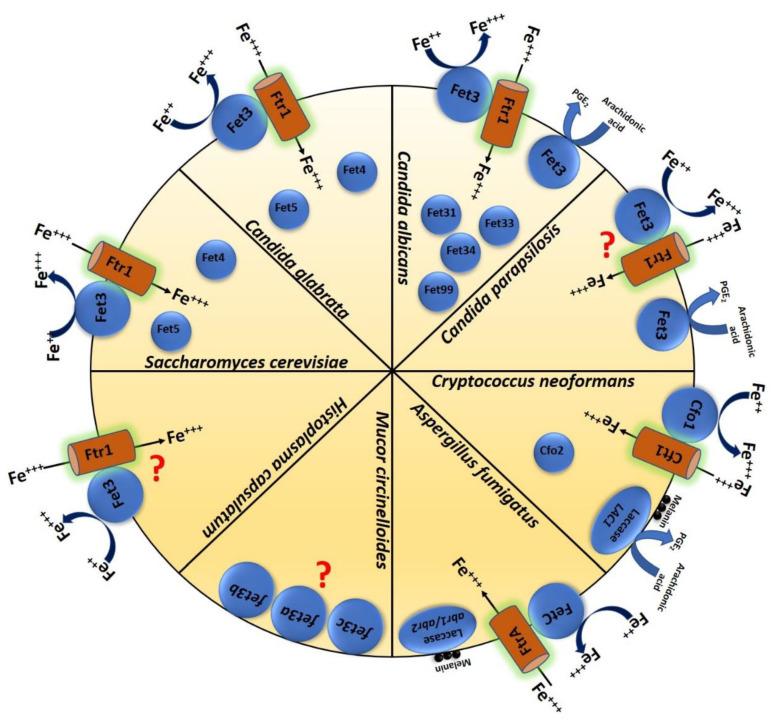
Schematic image showing important multicopper oxidases in different fungal species such as *S. cerevisiae*, *C. albicans*, *C. glabrata*, *C. parapsilosis*, *A. fumigatus*, *C. neoformans*, *M. circinelloides*, and *H. capsulatum* and their function.

Although not typically pathogenic in humans, studies in *S. cerevisiae* provide significant insights into MCOs in virulent fungi. Iron uptake is a two-step process in this species: (1) first, reduction of Fe (III) by iron reductases in the plasma membrane, followed by (2) the internalization of the Fe (II) products by either of two Fe (II) uptake systems. The plasma membrane-based Fe (III) reduction is a high-affinity system with a *K*m = 0.15 µM that requires *FET3* and is induced by low iron concentration. Fet3p is an essential component of the high-affinity reductive iron assimilation (RIA) pathway in fungi, which functions in combination with Ftr1p [20]. Fet3p functions similarly to human ceruloplasmin [21,22,23]. The internalization step is characterized by low affinity (*K*m = 30 µM) and requires Fet4p, which is generally active under iron-replete conditions [23]. Surprisingly, the *FET3-FET4* double deletion mutant of this species remains viable; however, it is extremely sensitive to iron limitation. This result led to the discovery of another ferroxidase in *S. cerevisiae,* namely *FET5*. Overexpression of this gene suppressed the growth defect of the *fet3Δ/Δ- fet4Δ/Δ* mutant under iron-limited conditions, confirming that Fet5p also plays a role in iron transport and homeostasis [24]. Fet3p forms a complex with the iron permease Ftr1p, a transmembrane protein. In *S. cerevisiae* (likely most fungi and plants), the extracellular Fe (III) is mobilized after the reduction to Fe (II) by the surface metalloreductase Fre1p and then oxidized by Fet3p. The oxidized form of Fe (III) is not a substrate of Ftr1p, so the iron uptake is paired strictly with ferroxidation. This permease-oxidase based transport system in *S. cerevisiae* provides a model that explains the copper-iron connection in biology [25,26]. Structural analysis shows that Fet3p belongs to the family of integral membrane protein (type 1). The N-terminal catalytic domain of the protein is located on the extracellular side of the plasma membrane and the single C-terminus transmembrane domain is tethered to the membrane [27]. The detailed 3D structure of the Fet3p extracellular ferroxidase domain shows a unique characteristic that enables Fe transport in eukaryotes. This structure also reveals structural differences between Fet3p and laccases [28,29]. Figure 1 schematically summarizes the presence of different MCOs in *S. cerevisiae* and various human pathogenic fungi.

## 3. Multicopper Oxidases and Their Function in Human Pathogenic Budding Yeast

### 3.1. Candida albicans

*C. albicans* is the most frequently isolated species of invasive *Candida* infections [30]. It is an opportunistic pathogenic fungus that is also a member of the healthy human mycobiota. It is able to cause both superficial infections of the skin or mucosal surfaces and invasive infections, where the fungus can spread to all vital organs through dissemination [31,32]. In order to survive within different host niches with restricted amounts of accessible iron, *C. albicans* has evolved various iron uptake mechanisms. Iron is an essential micronutrient for both the host and *C. albicans,* therefore iron uptake during infection also plays a role in the pathogenesis of this fungus [33]. Similar to *S. cerevisiae*, *C. albicans* has reductive iron uptake machinery and has several genes encoding ferric reductases (17 putative genes) and five ferroxidases belong to MCO in its genome [34]. 

The expression of the five ferroxidases, namely *FET3* (orf19.4211), *FET31* (orf19.4213), *FET33* (orf19.943), *FET34* (orf19.4215), and *FET99* (orf19.4212), varies depending on environmental conditions, including the availability of iron and oxygen and the presence of antifungals [35,36,37]. Functional characterization of these ferroxidases confirmed their role in iron uptake, morphological transition, and pathogenesis. A homozygous deletion mutant of *FET31* (referred to as *FET3* in the publication) showed a growth defect under low iron conditions; however, the mutant strain was as virulent as the wild type strain in a mouse model of systemic candidiasis [38]. *CaFET3* and *CaFET34* are important ferroxidases under iron starvation conditions. Specifically, deletion of *CaFET33* and *CaFET34* decrease the cellular iron content under iron limited conditions, although the mutants do not exhibit a growth defect. Deletion of *CaFET34* also significantly reduces filamentous growth and the virulence of *C. albicans* in a mouse model of systemic candidiasis [39]. Fet31p and Fet34p are localized to the plasma membrane and participate in Fe-uptake by forming a complex with Ftr1p [40]. Interestingly, *CaFET3* has also been associated with fungal prostaglandin E_2_ production [41].

### 3.2. Candida parapsilosis

*C. parapsilosis* is also an opportunistic human fungal pathogen, and depending on the country, it is the second or third most frequently isolated *Candida* species in immunocompromised patients with candidemia [42]. *C. parapsilosis* accounts for the highest numbers of candidiasis episodes in premature or low birth weight infants and can exist in diverse environments besides the human mycobiome. It typically exists in a yeast form, though it can generate pseudohyphae and it avidly forms multidrug resistant biofilms on abiotic surfaces such as dentures or catheters [42,43]. Although various virulence factors are associated with this fungus’ pathogenicity, the molecular mechanisms of iron metabolism and homoeostasis are poorly understood. Recently, however, three putative ferroxidase encoding genes were identified in its genome, which show high sequence similarity with *ScFET3*. A study of gene-deletion strains of CPAR2_603600 (79% identity with *CaFET3* and 54% identity with *ScFET3*) demonstrated that this gene is required for fungal growth under low iron conditions. The deletion mutant revealed a marked reduction in pseudohyphae and biofilm formation as well as alterations in the expression of many orthologous genes potentially involved in iron metabolism regulation (in press). Unlike in *C. albicans,* the deletion mutant had attenuated virulence in a mouse model of systemic candidiasis, suggesting a divergent role in these and possibly other species. Similar to *CaFET3,* this gene in *C. parapsilosis* also plays a role in PGE_2_ production from externally added arachidonic acids [44,45].

### 3.3. Candida glabrata

*C. glabrata* is also an opportunistic human fungal pathogen and part of the healthy human mycobiota [46]. *C. glabrata* is a unicellular budding yeast and phylogenetically closer to *S. cerevisiae* than to *C. albicans*. Approximately 12% of total bloodstream *Candida* infections globally are caused by *C. glabrata* and the mortality rate can reach up to 30% [46,47,48,49]. In silico analysis has revealed three ferroxidase genes, namely *CgFET3* (high affinity iron uptake; 70% similarity with *ScFET3*), *CgFET4* (low-affinity ferrous transporter of the plasma membrane; 58% similarity with *ScFET4*), and *CgFET5* (iron storage and utilization; 64% similarity with *ScFET5*). Phenotypic profiling of the *fet3Δ/Δ* deletion mutant in *C. glabrata* revealed that *FET3* is required for growth in the presence of the iron chelator bathophenanthrolinedisulfonic acid (BPS), suggesting that *FET3* is a part of the high-affinity iron uptake system in *C. glabrata*. The mutant strain is sensitive to oxidative stress and to the antifungal drug fluconazole, and also displays attenuated growth on media containing sodium chloride (osmotic stressor), caffeine and congo red (cell wall stressors), and SDS (sodium dodecyl sulfate, membrane stressor). Comparison of the intracellular iron levels reveals that the iron content of the *CgFET3* deletion mutant is ∼20%–50% lower than wild type cell. Disruption of this gene also resulted in ∼10%–20% reduction in the mitochondrial aconitase activity, which is a Fe-S containing protein [50]. *C. glabrata* is able to grow inside macrophages [51], but the deletion of *CgFET3* and *CgFET5* reduces this ability. Fungal burden analysis of the kidneys of mice revealed lower CFUs in mice infected with the mutant strains compared to the wild type [50]. Hence, *FET3* and *FET5* regulate the pathogenesis of *C. glabrata*. The homologue of an mRNA-degrading protein Cth2 has also been shown to regulate the expression *FET3* in *C. glabrata* [52,53].

### 3.4. Cryptococcus neoformans

Cryptococcosis remains a leading cause of death in the HIV/AIDS population as it is estimated that there are ~180,000 deaths annually, mostly in sub-Saharan Africa [54,55]. Most of these patients are either infected with *C. neoformans* or, to a lesser extent, *C. gattii* [54]. The two main components of the *C. neoformans* high affinity iron uptake system are the iron permease *CFT1* and the ferroxidase *CFO1*. Both *CFT1* and *CFO1* are present on chromosome 12 and are divergently transcribed. *C. neoformans* also contains *CFT2* and *CFO2*, which encode an iron permease and a ferroxidase, respectively, and are present on chromosome 3 [56]. Deletion mutants of each revealed that *CFO1* is required for high-affinity iron transport, however, the lack of *CFO2* led to no visible growth defect under limited iron conditions [57]. Using a mouse inhalation model of cryptococcosis, the virulence of the *cfo1Δ/Δ, cfo2Δ/Δ* and the *cfo1Δ- cfo2 Δ* double mutants was analyzed. The *cfo1Δ* single mutant as well as the *cfo1Δ- cfo2Δ* double mutant showed a significantly attenuated virulence compared to the wild type strain [58]. Similarly to the equivalent *C. glabrata* mutants, these mutant strains in *C. neoformans* were also more susceptible to fluconazole [58]. A localization study using GFP tagged *CFO1* revealed that the protein is localized to plasma membrane [59]. Interestingly, it has been suggested that the cAMP pathway regulates the intracellular trafficking of Cfo1p, as a defect in cAMP signaling leads to mis-localization of the Cfo1-GFP fusion protein [59]. 

*C. neoformans* also contains homologues of a laccase encoded by *LAC1* and *LAC2* expressed from the same chromosome. Deletion mutants of both the genes show significant reduction in melanin production [60]. The formation of melanin in the cell wall of this species protects the cell from different environmental stress conditions and host immune attacks [61]. The deletion mutant of these two genes also make the cells more susceptible to killing by alveolar macrophages [62]. The *LAC1* gene was also shown to be regulating fungal prostaglandin E_2_ production [63]. The *LAC1* gene also plays an important role in *C. neoformans*’ virulence as the mutant with the disrupted gene was not lethal in the mouse infection model [64].

## 4. Multicopper Oxidases and Their Function in Other Human Pathogenic Fungi

### 4.1. Mucor circinelloides

Mucormycosis is an emerging fungal infection threatening mainly immunocompromised patients suffering from diabetes or cancer or those underwent organ transplantation. The mortality rates of mucormycosis can reach as high as 90% in disseminated infections, which is a consequence of the lack of effective treatments and antifungal drug resistance [65,66,67,68]. *M. circinelloides,* a frequently used model to study mucormycosis, is a dimorphic fungus that can multiply through the formation of branching coenocytic hyphae under aerobic conditions or spherical multipolar budding yeasts in an oxygen deprived environment [69]. Three putative ferroxidase genes have been identified that show sequence similarity with *ScFET3: fet3a*, *fet3b,* and *fet3c.* Under aerobic conditions, only *fet3a* is expressed during the yeast phase, while the other two are specifically expressed in mycelia. Gene expression and deletion analysis of these genes revealed their necessity for growth under iron-limited conditions (media with iron chelator phenanthroline). Murine experiments with the *fet3aΔ/Δ*, *fet3bΔ/Δ* and *fet3cΔ/Δ* strains revealed that only fet3c significantly impacts virulence [70].

### 4.2. Histoplasma capsulatum

*Histoplasma capsulatum* is an intracellular pathogen that is the main cause of histoplasmosis in both immunocompetent and immunocompromised individuals. *Histoplasma* is a dimorphic fungus that is mycelial in nature and a yeast during mammalian disease [71]. Ohio and Mississippi river valleys, and the southeastern, central, and mid-Atlantic states report the most cases of histoplasmosis, and the infection rate can reach up to almost 500,000 individuals annually [72]. Although previous in silico analyses of the *H. capsulatum* G186AR genome identified orthologues of *S. cerevisiae FET3* and *FTR1* (sequence similarity: 65% for *FET3* and 61% for *FTR1*, respectively), functional studies have not yet been performed. Interestingly, in silico analyses showed that *H. capsulatum* strain (G217B) lacks the orthologues of either of these genes in the genome [73,74].

### 4.3. Aspergillus fumigatus

Invasive aspergillosis is the most common cause of airborne fungal invasive infections among immunocompromised patients worldwide and is primarily caused by *A. fumigatus* [75]. Patients with prolonged neutropenia are particularly susceptible [76]. The mortality rate of invasive aspergillosis is ~50% and increases in the setting of drug resistant strains [77,78,79,80]. Four iron uptake systems have been described in *A. fumigatus*: the low affinity ferrous iron uptake system (not yet characterized at the molecular level), two siderophore mediated high affinity ferric iron uptake systems, and the reductive iron assimilation system (RIA) [81]. Disruption of *sre*A, mediating siderophore biosynthesis in *A. fumigatus,* results in decreased virulence in mice [82,83]. The three major components of reductive iron assimilation are the ferric reductase FreB which helps in reduction of ferric to ferrous iron, then the import of ferrous iron through iron permease FtrA, and finally, oxidation by ferrous to ferric iron by ferroxidase FetC. FetCp is 52% identical to *C. albicans* Fet3p and FtrAp is 55% identical to *C. albicans* Ftr1p at the amino acid level [81,84] Although FetC is upregulated under iron-restricted conditions in *A. fumigatus*, a detailed characterization of this MCO has not been performed yet.

*A. fumigatus* contains two putative laccase encoding genes, namely Abr1 and Abr2, that are part of a gene cluster participating in melanin synthesis. The expression of *A. fumigatus* laccases Abr1/2 is dependent on hyphal competency and significantly increased during conidiation [85]. The *abr2Δ/Δ* mutant strain showed increased sensitivity to reactive oxygen species and reduced laccase activity in sporulating mycelia. However, unlike in *C. neoformans*, Abr2 deletion in *A. fumigatus* did not show any reduction in virulence in an intranasal mouse infection model [86]. Previous sequence analyses showed that the Abr1 protein does not contain the four residues that are present in *S. cerevisiae* Fet3 for Fe(II) binding, only the glutamic acid (E185) [87]. This also suggests that they represent a separate class of MCOs, evolved from an ancient canonical ferroxidase [88]. However, the deletion mutant of the *arp1* gene (regulating melanin production in *Colletotrichum lagenarium* and *Magnaporthe grisea*) in *A. fumigatus* produced reddish-pink conidia that are more susceptible to complement attack [89].

## 5. Conclusions

The family of MCOs is one of the most diverse family of enzymes, having a wide variety of functions. The importance of MCOs and their role in metal homoeostasis is well known in various human pathogens including bacteria, fungi, and parasites, although their detailed roles in virulence is still not fully explored. Multicopper oxidases are ubiquitous in the fungal kingdom which shows their evolutionary importance. In the current review, we aimed to collect all relevant information about different MCOs present in human fungal pathogens belonging to diverse fungal kingdoms, such as Ascomycetes, Basidiomycetes, and Zygomycetes. They have evolved different pathogenic mechanisms to infect human hosts. Our knowledge of the role of iron in fungal pathogenicity has advanced over recent years, however still little information is available about the precise role and inclusion of reductive iron uptake systems—including the Fet/Ftr complex—in pathogenicity mechanisms. For instance, the presence of iron for the human pathogenic *Paracoccidioides* species also influences virulence, although no detailed study is available about the MCOs of this particular fungus. Only in silico analyses suggest that PAAG_06004 and PADG_05994 could encode functional ferroxidases [90]. Laccases also play an important role in fungal pathogens by generating mainly melanin. However, more research is needed to address why some of the pathogenic fungi evolved a laccase enzyme system with the reductive iron system. Moreover, the presence of this evolutionary conserved system in pathogenic fungal species can be a potential target for selective therapeutic intervention in multiple mycoses.
